# Blocking heme oxygenase-1 by zinc protoporphyrin reduces tumor hypoxia-mediated VEGF release and inhibits tumor angiogenesis as a potential therapeutic agent against colorectal cancer

**DOI:** 10.1186/s12929-016-0219-6

**Published:** 2016-01-28

**Authors:** Chun-Chia Cheng, Siao-Syun Guan, Hao-Jhih Yang, Chun-Chao Chang, Tsai-Yueh Luo, Jungshan Chang, Ai-Sheng Ho

**Affiliations:** Institute of Nuclear Energy Research, Atomic Energy Council, Taoyuan, Taiwan; Institute of Clinical Medicine, National Yang-Ming University, Taipei, Taiwan; Division of Gastroenterology and Hepatology, Department of Internal Medicine, Taipei Medical University Hospital, Taipei, Taiwan; Department of Internal Medicine, School of Medicine, College of Medicine, Taipei Medical University, Taipei, Taiwan; Graduate Institute of Medical Sciences, School of Medicine, College of Medicine, Taipei Medical University, Taipei, Taiwan; Division of Gastroenterology, Cheng Hsin General Hospital, Taipei, Taiwan; Nursing Department, Kang-Ning University, Taipei, Taiwan

**Keywords:** Angiogenesis, Heme oxygenase-1, Tumor hypoxia, Vascular endothelial growth factor, Zinc protoporphyrin

## Abstract

**Background:**

Hypoxia in tumor niche is one of important factors to start regeneration of blood vessels, leading to increase survival, proliferation, and invasion in cancer cells. Under hypoxia microenvironment, furthermore, steadily increased hypoxia-inducible factor-1α (HIF-1α) is observed, and can increase vascular endothelial growth factor (VEGF) expression and promote angiogenesis. Zinc protoporphyrin (ZnPP), a heme oxygenase-1 (HO-1) inhibitor, is potential to inhibit tumor proliferation and progression. However, the mechanism of ZnPP in inhibition of tumor is not completely clear. We hypothesize that ZnPP may modulate HIF-1α through inhibiting HO-1, and then inhibit angiogenesis and tumor progression. This study aimed to dissect the mechanism of ZnPP in tumor suppression.

**Results:**

We observed the amount of VEGF was increased in the sera of the colorectal cancer (CRC) patients (*n* = 34, *p* < 0.05). Furthermore, increased VEGF expression was also measured in colorectal cancer cells, HCT-15, culturing under mimicking hypoxic condition. It suggested that hypoxia induced VEGF production from cancer cells. VEGF production was significantly reduced from HCT-15 cells after exposure to HIF-1α inhibitor KC7F2, suggesting that HIF-1α regulated VEGF production. Moreover, we observed that the HO-1inhibitor ZnPP inhibited the expressions of HIF-1α and VEGF coupled with cell proliferations of HCT-15 cells, suggesting that ZnPP blocked HIF-1α expression, and then inhibited the consequent VEGF production. In the xenograft model, we also observed that the animals exposed to ZnPP displayed much smaller tumor nodules and less degree of angiogenesis with decreased expression of the angiogenesis marker, αvβ3 integrin, compared to that in normal control.

**Conclusions:**

This study demonstrated that VEGF level in serum was elevated in the patients with CRC. The HO-1 inhibitor, ZnPP, possessed the properties of anti-tumor agent by decreasing HIF-1α levels, blocking VEGF production, impairing tumor angiogenesis, and inhibiting tumor growth.

## Background

In the process of tumor progression, the content of oxygen in blood is one of important factors contributes to proliferations, angiogenesis and metastasis in solid tumors. Due to highly accelerated cell divisions and proliferations, tumor cells survive in the microenvironments with deprived oxygen termed as hypoxia, in which may elicit signals to form new blood vessels around tumors for providing additional oxygen and nutrient to tumors [1]. Under this hypoxia condition, deprivation of oxygen in blood causes pathophysiologic consequences within tumor cells, leading to exacerbate tumor progression, tumor invasion and gain the resistance to apoptotic cell death program [2, 3]. In addition to increased capability in progression, invasion, and proliferation, hypoxia may also strength tumor cells with higher resistance to radiotherapy and chemotherapy [4, 5]. Hypoxia-inducible factor-1α (HIF-1α), a regulatory transcription factor, plays a crucial role for tumor cells in responding to lower oxygen in their resident microenvironment. Increased HIF-1α induced by hypoxia can commit the adaptive changes in gene expressions of tumor cells [2, 6, 7]. It suggests that HIF-1α is one of potential therapeutic targets in tumors.

Currently, zinc protoporphyrin (ZnPP), a heme oxygenase-1 (HO-1) inhibitor, is one of therapeutic candidates of tumors [8, 9]. It has been documented that the amount of HO-1 is elevated under hypoxic condition, and this phenomena is mediated by HIF-1α [10–12]. The physiological function of HO-1 is to cleave heme and give products such as carbon monoxide (CO), iron ion, and biliverdin [12]. CO and bilirubin derived from biliverdin are antioxidants which may benefit cells in growing and surviving. It implies that HO-1 is a cytoprotective enzyme [13–16]. In addition, other studies also indicate that HO-1 can trigger and regulate HIF-1α expression in hypoxic tumor cells [17]. The overexpressed HO-1 seems to link with increased tumor growth and drug resistance to chemotherapeutic agents [3, 18].

Since HO-1 is associated with tumor progression, it may be as another therapeutic target in treatment of cancers [12, 19] such as colorectal cancer (CRC) [20]. Previous studies have indicated that the drugs by inhibition of HO-1 are considered to be potential therapeutic candidates in the treatment of cancers [21, 22]. ZnPP is well known as an inhibitor of HO-1 and can reduces cancer cell growth. However, this ZnPP-induced inhibitory effect on cancer cell growth is suggested as HO-1 independent manner by Wong et al. [23]. This result suggests that ZnPP may be through other unknown mechanism to inhibit tumor growth other than the known mechanism by inhibition in HO-1 activity. In addition to be an inhibitor of HO-1, ZnPP also acts as an enzymatic substrate of HO-1. Due to the similarity in structure between ZnPP and heme, ZnPP can compete with heme to HO-1, leading to reduce the productive levels of CO and bilirubin [24], which may interfere, disrupt, or detour other cellular signaling pathways to suppress the growth of tumors [23]. However, the detail anti-tumor mechanism of ZnPP for the CRC therapy is still unclear and need to be evaluated. Therefore, this presented study was to investigate the anti-tumor functions and molecular/cellular mechanisms of ZnPP in CRC.

It has been demonstrated that tumor hypoxia induces HO-1 expression [10], and also increases the production of vascular endothelial growth factor (VEGF) [6, 25–27]. VEGF is a ligand of VEGF receptor expressed on the endothelial cells, which triggers the signaling for angiogenesis. Since HO-1 may be associated with the expression of HIF-1α and VEGF [28], we hypothesized that HO-1 inhibitor ZnPP was able to obstruct tumor growth and spread of cancer cells by inhibiting the HO-1-induced HIF-1α expression coupled with VEGF-mediated angiogenesis. In order to evaluate this hypothesis, we treated human colorectal carcinoma cells (HCT-15) with ZnPP, and then measured the amount of HO-1, HIF-1α and VEGF expression level. Furthermore, we also evaluated the therapeutic benefits in HCT-15-induced tumor xenografts after treatment of ZnPP such as in the size/volume of tumors and the degree of angiogenesis.

## Methods

### Sera and tissues from the patients with colorectal cancer

The clinical samples including sera and tissues were collected from Cheng Hsin General Hospital, Taiwan, which was approved by the Institutional Review Board (CHGH-IRB-(240) 100–01). The pairs of tissues including tumors (T) and adjacent non-tumors (NT) from the CRC patients were acquired by surgery. We only collected and analyzed the type of colorectal adenocarcinoma. Total 34 pairs of clinical tissues from the enrolled patients were stained using methylene blue staining and distinguished by a pathologist. Tumor histopathology and severity were determined according to the rules of American Joint Commission on Cancer Staging (AJCCS) system. The 15 healthy volunteers enrolled in this study had no evidence of known CRC. Surgery for identifying normal phenotype was not performed in the healthy volunteers due to ethical issues.

### HCT-15 culture and tumor xenograft model

Human colorectal carcinoma cells (HCT-15) were cultured in F12K medium with 10 % of fetal bovine serum. All cells were incubated at 37 °C and 5 % CO_2_. Male nude mice were purchased from BioLASCO Taiwan Co., Ltd, Taiwan. The 5-week-old mice were housed in a 12 h light cycle at 22 °C. The animal studies were approved by the institutive ethical review committee in Institute of Nuclear Energy Research, which followed the NIH guidelines on the care and welfare of laboratory animals. HCT-15 cells (2 × 10^6^) were subcutaneously (s.c.) inoculated into the right leg of nude mice. Tumors were established for 10 days before the tumor treatment and imaging.

### Tumor hypoxia

For control group, HCT-15 cells were cultured in the normal condition at 37 °C and 5 % CO_2_. To mimic a hypoxic condition, HCT-15 was incubated at 37 °C with 21 % CO_2_ in an anaerobic incubator (Mitsubishi Gas Chemical, Tokyo, Japan). HCT-15 cells were harvested for detecting the carbonic anhydrase 9 (CA9), HO-1, and β-actin expressions using Western blots when HCT-15 cells were incubated in 24 h and 48 h.

### Tumor inhibition *in vitro*

HCT-15 cells with 50 % confluence were cultured in F12K medium with 2.5 μM or 10 μM of hemin and ZnPP for 24 h. The tumor cells without treatment were used as the experimental controls. The cell numbers were calculated using an automatic cell counter (Invitrogen, Massachusetts, USA). Moreover, the cells treated with or without 10 μM of hemin and ZnPP were collected and analyzed (*n* = 3) using Western blots for detecting HIF-1α, HO-1, glucose regulated protein 78 (GRP78), and β-actin. Apoptosis was evaluated by Annexin V-FITC staining. The cells were washed and harvested for flow cytometric analysis in a FACSCalibur Flow Cytometer (BD Biosciences, New Jersey, USA).

### VEGF measurement

VEGF levels were measured using a VEGF enzyme-linked immunosorbent assay (ELISA) kit (Invitrogen, Massachusetts, USA). There were three conditions where the mediums were collected and measured. The mediums were collected where (1) HCT-15 cells with 90 % confluence were cultured in normoxia or hypoxia condition, (2) HCT-15 cells with 90 % confluence were treated with 2.5 μM or 10 μM of hemin and ZnPP, and cultured in normoxia condition for 24 h, and (3) HCT-15 cells with 90 % confluence were treated with 2.5 μM or 10 μM of hemin and ZnPP, and cultured in hypoxia condition for 24 h. VEGF measurements were performed according to the manufacturer’s instructions and quality control was ensured.

### Western blots

Cells were lysed in the buffer containing 150 mM NaCl, 1 % NP-40, 0.1 % SDS, and 50 mM Tris–HCl (pH8.0). The protein samples were mixed with two-fold sample buffer (75 mM of Tris–HCl, pH 6.8, 10 % (v/v) glycerol, 2 % SDS (w/v), 0.002 % (w/v) bromophenol blue). Total 20 μg of each sample was analyzed in 10 % sodium dodecyl sulfate polyacrylamide gel electrophoresis, and then transferred onto the Immobilon P membranes (Merck Millipore, Massachusetts, USA). These PVDF membranes were blocked in 3 % skim milk for 1 h at room temperature. PVDF membranes were then incubated with primary antibodies (1 μg/ml) overnight at 4 °C, and washed using Tris buffered saline with 0.1 % tween-20. After washing, PVDF membranes were incubated with horseradish peroxidase-conjugated secondary antibody (1 μg/ml) for 2 h at room temperature. The immunoreactive proteins were detected using ECL (enhanced chemiluminescence, Bio-Rad, California, USA) coupling with a LAS-4000 mini device (Fujifilm, Tokyo, Japan).

### Angiogenesis imaging *in vivo*

The PBS (*n* = 3) or 100 μg of ZnPP (*n* = 3) were injected into HCT-15 tumor xenografts from tail vein. After 24 h circulation, the angiogenesis detecting agent labeled with the near-infrared fluorescence (*AngioSense* 680 EX, PerkinElmer, Massachusetts, USA) was intravenously injected into tail vein of HCT-15 xenograft model. Then, the *in vivo imaging system* (IVIS, PerkinElmer, Massachusetts, USA) was performed to capture the fluorescent images for detecting angiogenesis distribution.

### Inhibition of tumor growth by ZnPP *in vivo*

For testing the anti-tumor effects of ZnPP, the mice were randomly divided to three groups: PBS as control (*n* = 3), 10 μg of ZnPP (*n* = 3), and 100 μg of ZnPP (*n* = 3). The mice were treated in day 11, 13, and 16 after tumor implantation. The tumor sizes were measured using a digital caliper and recorded in day 11, 13, 16, 18, and 20. Tumor volumes were recorded and calculated using the formula: 0.52 x width^2^ x length, herein the width represents the smaller tumor diameter. The mice were sacrificed in day 20, and tumors were analyzed using Western blots for detecting αvβ3 integrin and β-actin.

### Statistical analysis

The bands in Western blots were quantified by densitometric analysis using Multi Gauge v3.2 software (Fujifilm, Tokyo, Japan). Statistical analysis was performed using GraphPad Prism V5.01 software (GraphPad Software, Inc., California, USA). All analysis data with more than two groups were performed by ANOVA followed by post-hoc analysis using Bonferroni’s test. Student’s *t* test was used to compare two groups. The analysis of receiver operating characteristic curve was performed to clarify the cut-off value using SPSS software. Data were presented as mean ± SD. The significance difference (*p* value) was acceptable as *p* < 0.05.

## Results

### VEGF elevated in the sera of CRC patients

**Fig. 1:**
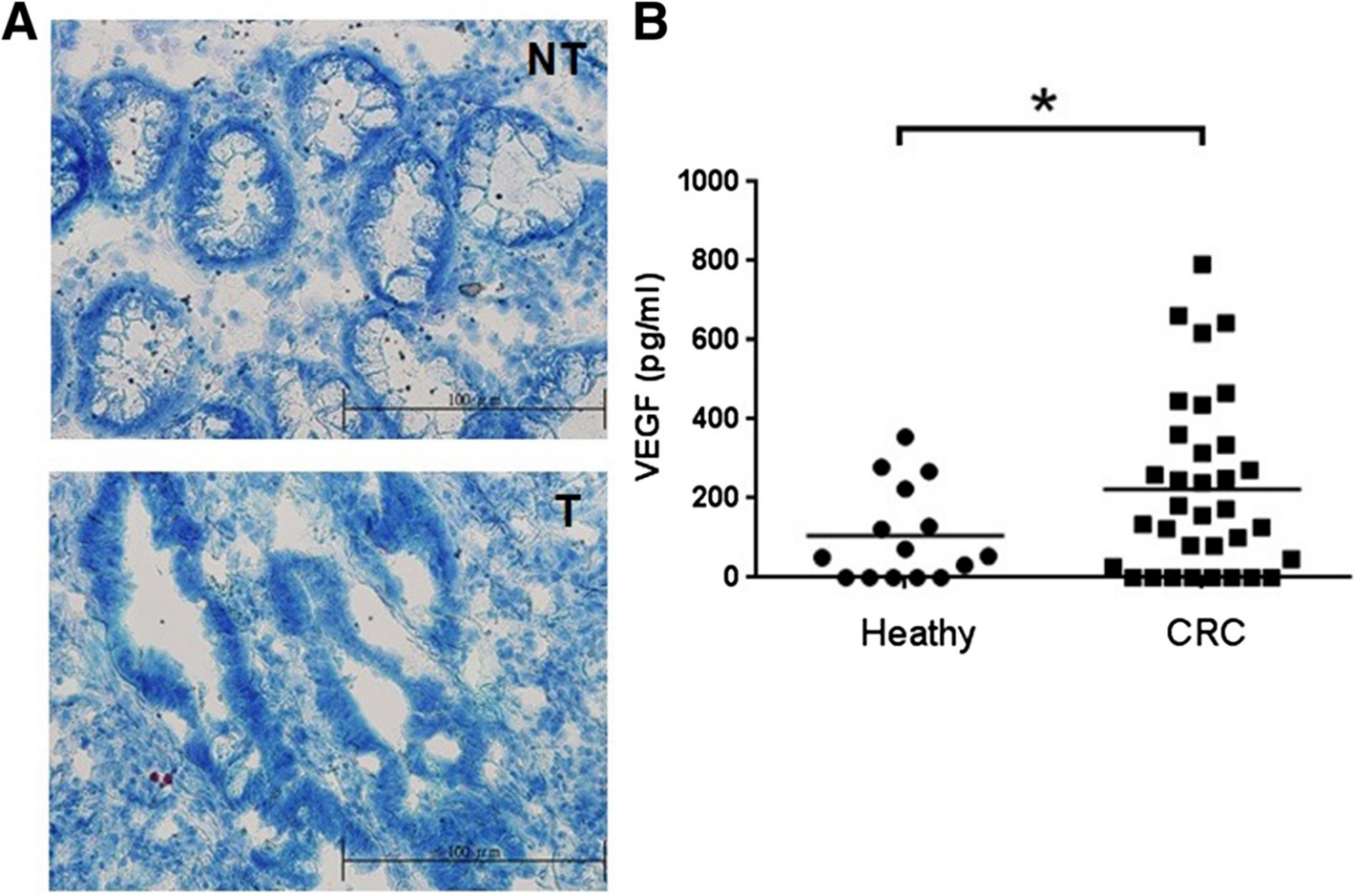
VEGF overexpressed in the sera of the enrolled patients with colorectal cancer. **a** Total 34 pairs of clinical tissues from the enrolled patients were stained using methylene blue staining and distinguished by a pathologist. The healthy volunteers were not tested due to ethical issues, who had no evidence of known CRC. NT: non-tumor; T: tumor. Scale bar: 100 μm. **b** VEGF levels were increased in the sera of patients with colorectal cancer (*n* = 34) compared to that in healthy volunteers (*n* = 15). *p < 0.05

To compare VEGF levels in sera between the CRC patients and the normal individuals, sera samples were acquired and measured using the ELISA assay. Thirty-four CRC patients and 15 healthy volunteers were enrolled and participated in this study. The tumor tissues were first collected by surgical operation and then stained using methylene blue dye for distinguishing tumors from the adjacent non-tumor tissues in each clinical biopsy specimens (Fig. 1a). Due to the medical ethics, the normal tissues from the healthy volunteers were not collected. The serological VEGF levels in CRC group were 222 ± 38 pg/ml compared to that in healthy group measured as 105 ± 31 pg/ml (Fig. 1b), indicating the significantly increased VEGF expression in sera of CRC patients. Moreover, the sensitivity and specificity of VEGF were 0.618 and 0.667, respectively, with a cut-off value of 122.5 pg/ml according to the analysis of receiver operating characteristic curve.

### Tumor hypoxia promoted cell growth and enhanced HO-1 and VEGF expression in HCT-15 cells

**Fig. 2:**
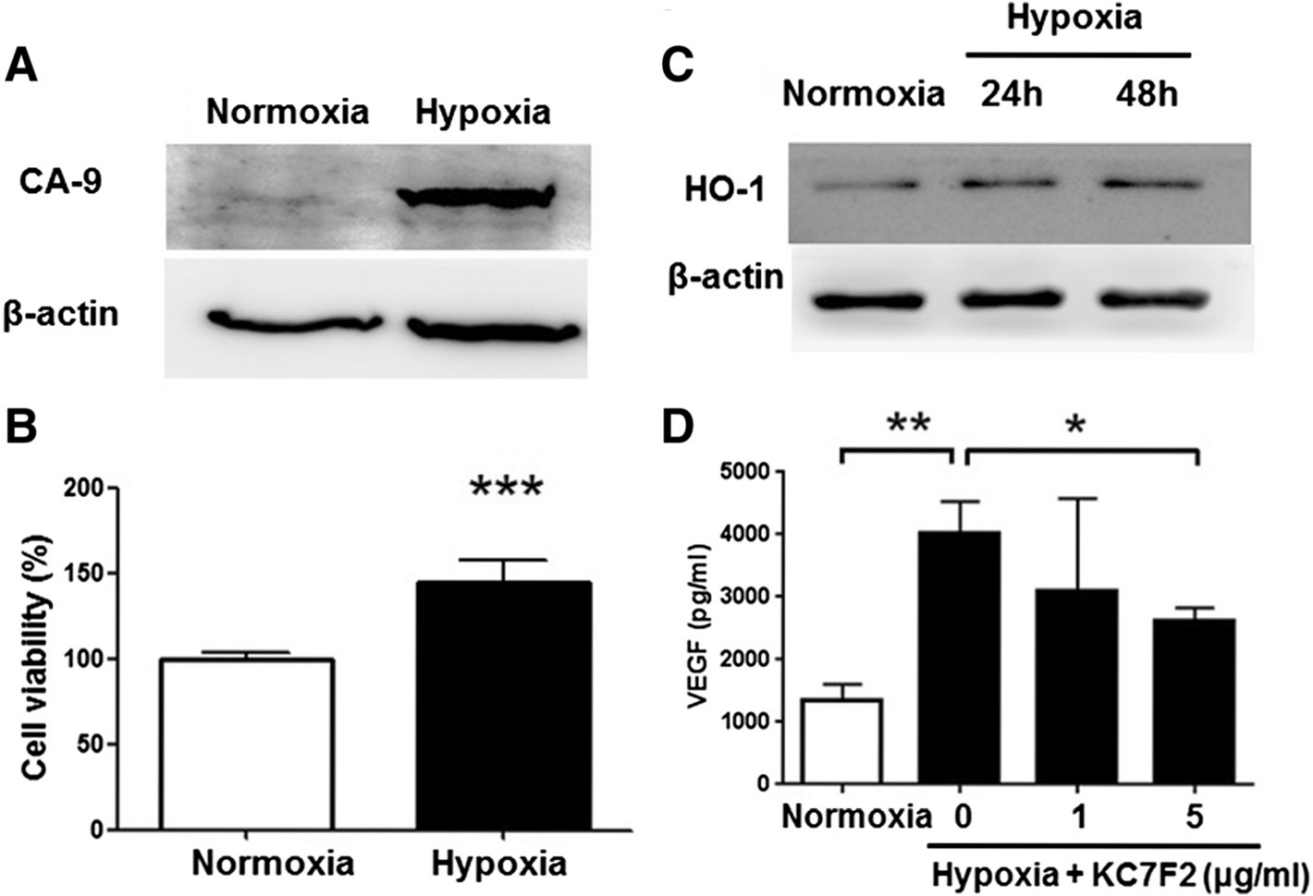
Tumor hypoxia elevated HO-1 and VEGF levels. **a** Tumor hypoxia marker, carbonic anhydrase IX (CA9), increased in the condition mimicking hypoxia compared to normoxia. **b** Cell viability was increased when HCT-15 cells were cultured in hypoxic condition for 24 h compared to nomoxia. **c** HO-1 overexpressed in mimic tumor hypoxia condition compared to that in normoxia detected using Western blots. **d** The secreted VEGF elevated in tumor hypoxia compared to that in normoxia. In addition, a HIF-1α inhibitor, KC7F2, was used to block the HIF-1α function, leading to decrease VEGF levels, revealing that VEGF overexpressed in tumor hypoxia, and was regulated by HIF-1α. *p < 0.05. **p < 0.01. ***p < 0.001

To elevate the role of hypoxia on cell survival, HCT-15 cells were cultured on hypoxic incubator supplemented with 21 % CO_2_ as described in the section of Methods and Materials. HCT-15 cells growing under hypoxic condition displayed significantly higher amount of carbonic anhydrase-9 (CA9), a hypoxic marker, compared to cells growing under normoxia condition (Fig. 2a). Furthermore, HCT-15 cells culturing in hypoxic condition gained more than 50 % in cell viability compared to normoxia (Fig. 2b). Since HIF-1α was more stable with longer half-life in activity under hypoxia, leading to promote tumor angiogenesis [6, 29], we further measured the expressions of HO-1 and VEGF in HCT-15 cells culturing in hypoxic condition. To measure the changes of HO-1 levels expressed from HCT-15 growing under normoxia and hypoxia, cell protein lysates were acquired and then detected using Western blots. The detection of VEGF secreted from HCT-15 into cultured medium was measured using ELISA. We found that HO-1 expression was significantly increased in HCT-15 cells cultured either for 24 h or 48 h in hypoxic condition (Fig. 2c). Moreover, the amounts of VEGF in cell supernatant derived from cells culturing under hypoxia and normoxia were measured and the results revealed more than 3-fold increase in VEGF production from cells grew under hypoxic condition (Fig. 2d). Moreover, the hypoxia-induced VEGF production from HCT-15 cells was blocked by KC7F2, a HIF-1α inhibitor. These results suggested that HIF-1αplayed an important role on regulation to induced HO-1 and VEGF expressions.

### ZnPP inhibited tumor proliferation coupling with reduced HIF-1α and HO-1 expressions

**Fig. 3:**
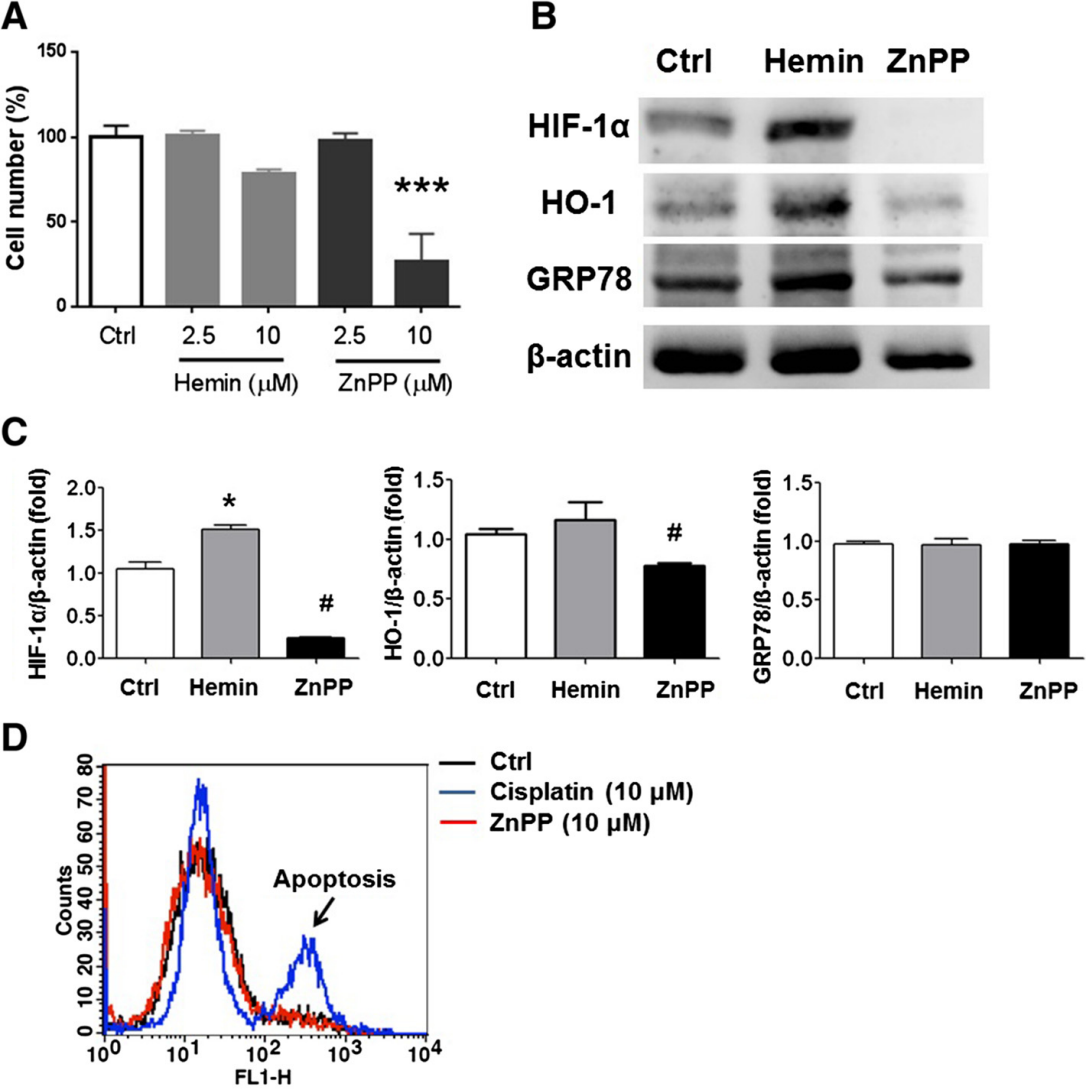
ZnPP inhibited cell proliferation and decreased HIF-1α levels in HCT-15 cells. **a** HCT-15 cells cultured in normoxia were treated with hemin, a HO-1 inducer, or ZnPP, a HO-1 inhibitor, using a dose-dependent manner, and the cell number was counted and compared. The cells without treatment were used as control (Ctrl). ZnPP (10 μM) significantly inhibited the HCT-15 cell viability, but hemin did not. **b** & **c** The treated cells were harvested and investigated (*n* = 3) for detecting the expressions of HO-1, HIF-1α, ER stress marker GRP78, and β-actin. Hemin increased HIF-1α and HO-1 (without significance), but ZnPP decreased HIF-1α and HO-1. The ratio of GRP78/β-actin was equivalent among the groups as an indicator excluding the treated effects derived from hemin or ZnPP. **d** Furthermore, apoptosis was not detected in HCT-15 cells treated with 10 μM of ZnPP, whereas 10 μM of cisplatin was used as a positive control to induce apoptosis, indicating that ZnPP inhibited HCT-15 cell proliferation without leading to apoptosis. *p < 0.05. ***p < 0.001. ^#^ *p* < 0.05 compared to ctrl

Since hypoxia-induced HO-1 was observed in tumor cells, we hypothesized that HO-1 may act as a therapeutic target for treatment of cancers. ZnPP is a competitive HO-1 inhibitor for competing with heme. Therefore, we investigated the inhibitory roles of ZnPP on survival of HCT-15 cells. HTC-15 cells were cultured with medium containing 2.5 or 10 μM and the result demonstrated that numbers of HCT-15 cells were significantly decreased with ~75 % reduction at 10 μM of ZnPP (*p* < 0.05) but no cytotoxic effect at 2.5 μM. Moreover, hemin, a HO-1 inducer, did not statistically alter the numbers of cells either at 2.5 or 10 μM of hemin (Fig. 3a). In order to detail the cytotoxic mechanism of ZnPP on HCT-15 cells, we analyzed and measured several candidate proteins in expressions level such as HO-1, HIF-1α, and endoplasmic reticulum (ER) stress GRP78 in HCT-15 cells cultured in medium either containing 10 μM of hemin or 10 μM of ZnPP. The results indicated that hemin significantly increased amounts of HIF-1α and HO-1, although no statistically significance in hemin-enhanced HO-1 production. In contrast to hemin, ZnPP significantly reduced HIF-1α and HO-1 expressions (Fig. 3b & c). Both hemin and ZnPP did not affect the expression of GRP78, an ER stress protein overexpressed under extreme conditions countering to non-specific stimulations [30]. To characterize the potential mechanism of ZnPP-induced reduction in cell numbers, the apoptotic analysis was performed and the results demonstrated that ZnPP at 10 μM did not trigger apoptotic program in HCT-15 cells (Fig. 3d). We presumed that ZnPP may reduce HIF-1α expression through inhibiting HO-1 activity, and consequently inhibit tumor proliferation.

### ZnPP reduced hypoxia-mediated VEGF release in HCT-15 cells *in vitro*

**Fig. 4:**
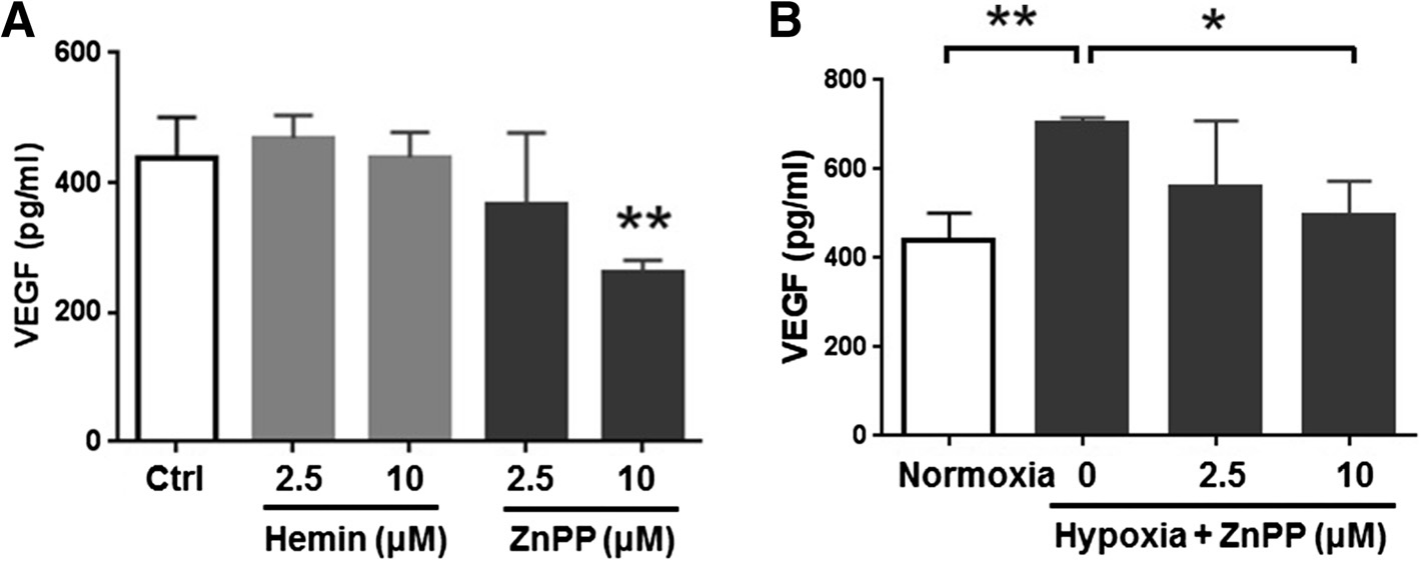
ZnPP reduced tumor hypoxia-mediated VEGF release. **a** HCT-15 cells were treated with hemin or ZnPP cultured in normoxia, and the cultured mediums were collected and investigated using ELISA assay. We found that ZnPP (10 μM) decreased VEGF secretion in HCT-15 cells, but hemin did not. **b** HCT-15 cells was treated with ZnPP and cultured in hypoxia condition. Tumor hypoxia increased VEGF, but ZnPP reduced hypoxia-induced VEGF levels. *p < 0.05. **p < 0.01

Our data demonstrated that hypoxia mediated HO-1 production and VEGF release from HCT-15 cells, ZnPP, however, inhibited HIF-1α expression. Therefore, we were interested in evaluating the effects of ZnPP on VEGF expression in HCT-15 cells. To collect and measure the amounts of VEGF in supernatant of cultured medium, the HCT-15 cells were cultured in medium containing either with hemin or ZnPP at concentration of 2.5 or 10 μM for 24 h incubation. VEGF levels were then measured using ELISA assay. The results showed that ZnPP at 10 μM significantly reduced VEGF levels by 40 % (262 ± 18 pg/ml) compared to control group without any treatments (438 ± 62 pg/ml). Meanwhile, hemin displayed no any modulating effects on VEGF production in HCT-15 cells either at 2.5 or 10 μM (Fig. 4a). This result indicated that ZnPP specifically reduced VEGF release from HCT-15 cells. Since tumor hypoxia specifically induced VEGF expression and release as shown in Fig. 2d, we would like to see whether ZnPP can interfere hypoxia-induced VEGF production or not. We cultured cells under hypoxic chamber either with or without treatments of ZnPP and then the measurements of VEGF were acquired and compared. The results first confirmed that hypoxia induced VEGF production from HCT-15 cells. Furthermore, ZnPP inhibited hypoxia-induced VEGF production in a dose-dependently manner (Fig 4b).

### ZnPP reduced tumor angiogenesis and tumor growth in HCT-15-induced xenografts

**Fig. 5:**
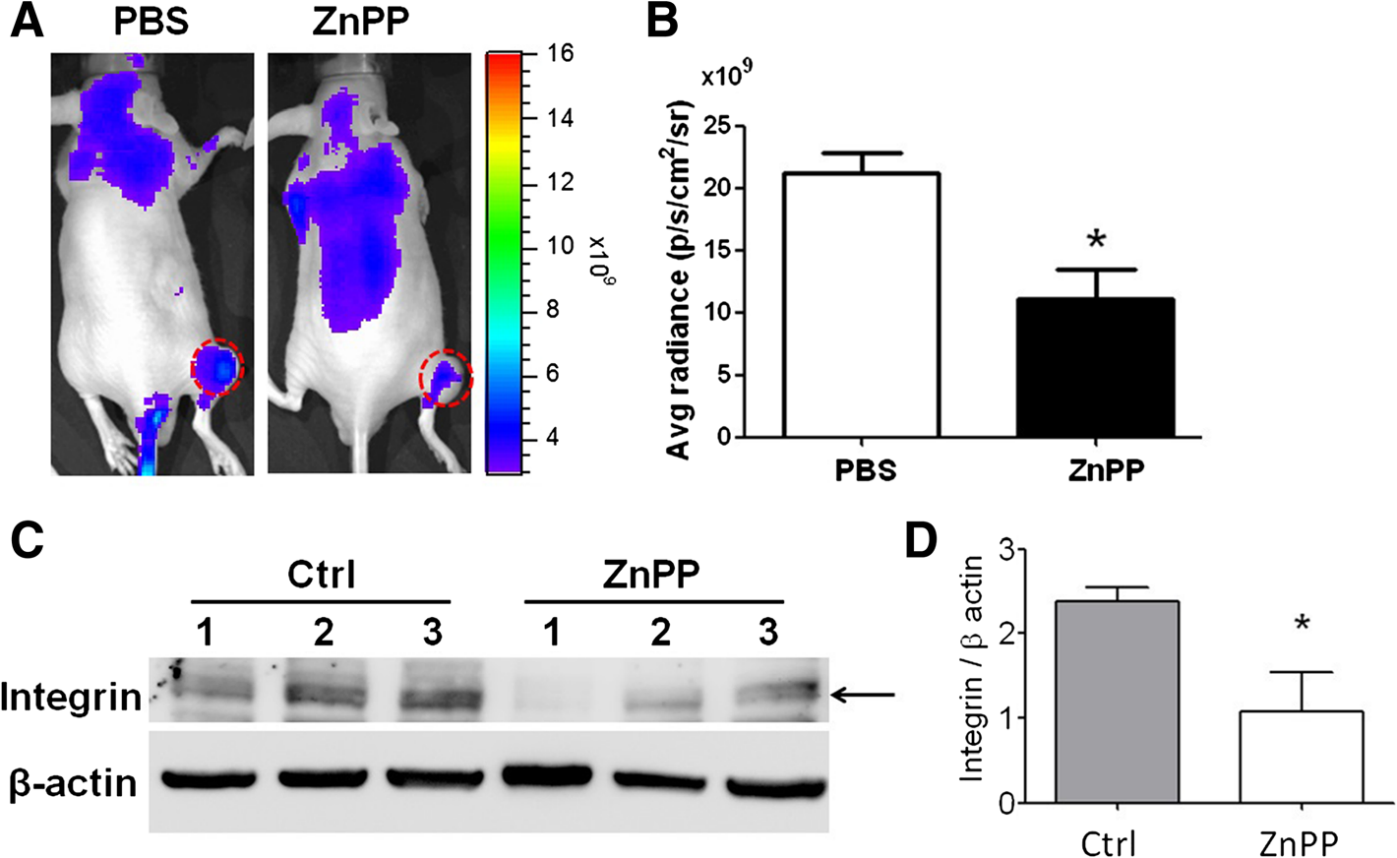
ZnPP inhibited tumor angiogenesis in the HCT-15-induced tumor xenografts. **a**-**b** The mice were injected with PBS or 100 μg of ZnPP through tail vein three times (day 11, 13, and 16), and the angiogenesis fluorescent agent was injected for measuring the angiogenesis levels within tumors. The fluorescent intensity per square measure in the area of circle decreased by ~50 % in tumors treated with ZnPP compared to that treated with PBS. Tumors were indicated by circles. **c**-**d** The tumors from HCT-15-induced xenografts treated with PBS or ZnPP were investigated and compared using Western blots. The angiogenesis marker, αvβ3 integrin, decreased in the tumors treated with ZnPP compared to PBS by ~50 %. *p < 0.05

We demonstrated that ZnPP-mediating reduction in HCT-15 proliferation combined with decreased VEGF release, so we further liked to investigate the *in vivo* inhibitory potential of ZnPP on tumor and angiogenesis in animals suffered with cancers. VEGF are positively associated with angiogenesis during progression of cancers. To characterize the inhibitory potential of ZnPP in angiogenesis, ZnPP was intravenously administrated into xenografts with HCT-15 cancer cells and then angiogenesis were detected and analyzed using an *in vivo* imaging system (IVIS) coupled with a near-infrared labeled fluorescent macromolecule (*AngioSense* 680 EX, Perkin Elmer, Massachusetts, USA) via intravenous administration. The results indicated that less fluorescence-labeled area combined with lower fluorescent intensity (~50 % reduction) in the animals pretreated with ZnPP compared to that in animals only treated with PBS (Fig. 5a and b), revealing that ZnPP reduced tumor angiogenesis *in vivo*. In order to confirm the results derived from imaging analysis by IVIS, we measured the amount of αvβ3 integrin using Western blotting. According to the literatures, αvβ3 is one of angiogenetic markers [31, 32]. The αvβ3 integrin expressed from ZnPP-treated tumor tissues were significantly reduced by 50 % compared to that in PBS treatment (Fig. 5c and d). The decreased ratio of αvβ3 integrin was consistent with that our previous observation in IVIS detection of tumor angiogenesis. These results indicated that ZnPP inhibited tumor angiogenesis and this ZnPP-mediated anti-angiogenesis effect was VEGF dependent.

**Fig. 6:**
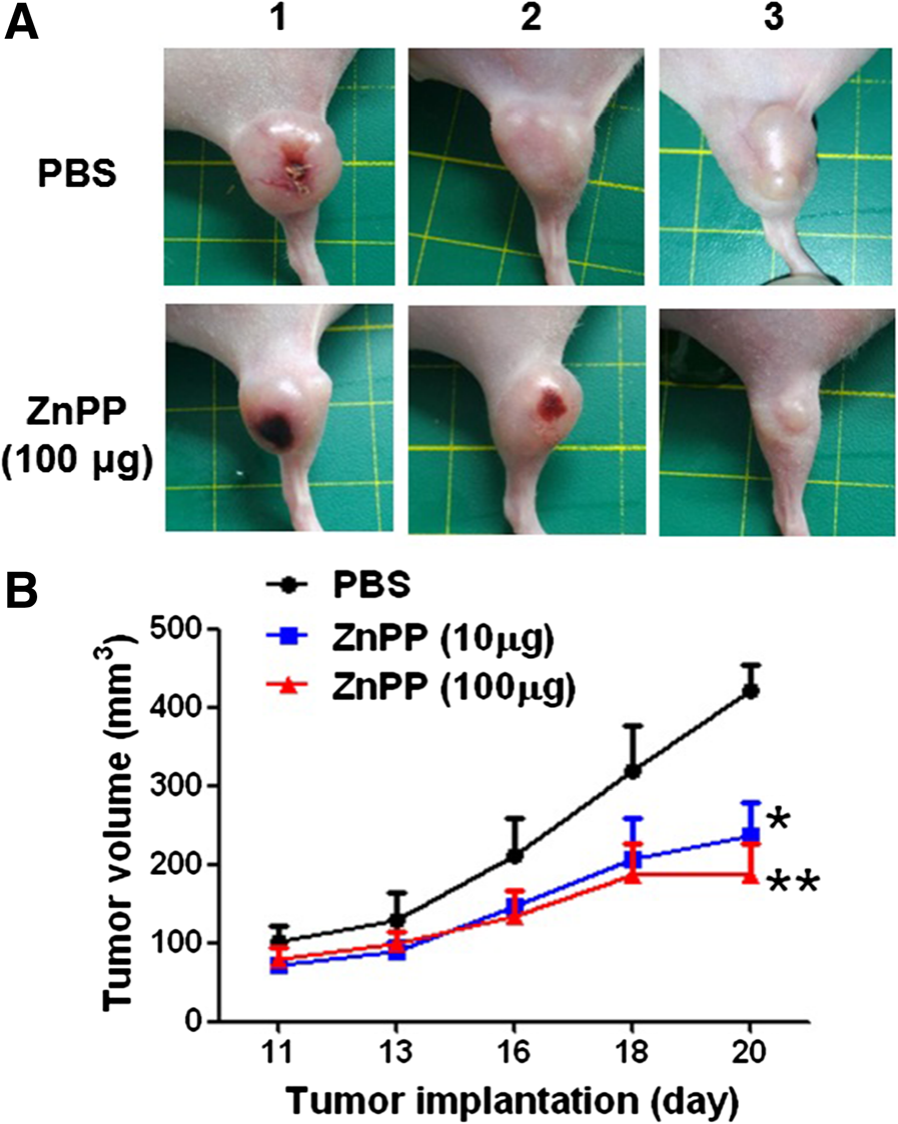
ZnPP inhibited tumor growth in the HCt-15-induced tumor xenografts as a potential therapeutic agent. **a** The mice were treated with PBS, 10 μg or 100 μg of ZnPP three times (day 11, 13, and 16), and the tumor sizes were recorded and compared. We found that 100 μg of ZnPP significantly reduced the tumor growth, and **b** decreased tumor volume by ~50 %. *p <0.05. **p <0.01

Beside the role of ZnPP on anti-angiogenesis, we also would like to evaluate the therapeutic effects of ZnPP on HCT-15-induced xenografts. Since ZnPP was demonstrated to reduce VEGF-triggered angiogenesis, ZnPP was injected into HCT-15-induced xenografts through tail vein at the day 11, 13, and 16 after tumor implantation. We found that both concentration at 10 μg and 100 μg of ZnPP significantly reduced the tumor size compared to PBS group (Fig. 6a and b). The results indicated that ZnPP may be a potential therapeutic candidate for patients with colorectal cancer.

## Discussion

ZnPP, one of metalloporphyrins, is a HO-1 inhibitor by competing metabolized heme. A previous study suggests that tin protoporphyrin IX (SnPP) is the most potent HO-1 inhibitor in the internal anal sphincter (IAS) smooth muscle [33]. Literatures also have indicated that other inhibitors but not SnPP show strong inhibitory function on HO-1 activity in liver [33]. It suggested the HO-1 inhibitors are belonging to tissue-specific inhibitors. Compared to other metalloporphyrins such as copper protoporphyrin (CuPP), ZnPP displays more potent inhibitory function on HO-1 activity in tumor [34], indicating that ZnPP may be a good candidate to inhibit growth and progression of tumors. A particular study indicates that ZnPP suppresses cyclin D1 gene expression in cancer cells is HO-1 independent, but SnPP does not [35]. Another study demonstrated that ZnPP-induced tumor suppression effect is a HO-1-independent manner, but via in inhibiting the Wnt/β-catenin signaling pathway in cancer cells [36]. Thus, it is likely that ZnPP, a HO-1 inhibitor, not only reduces the HO-1 activity, but also triggers other inhibitory effects on other mechanism associated with tumor cell progression.

In our current study, we found that ZnPP prohibited cell proliferation in HCT-15 cells, decreased HIF-1α and HO-1 levels, reduced VEGF release, and inhibited angiogenesis. Tumor hypoxia prolongs HIF-1α activity, and induces VEGF expression, leading to promote angiogenesis and malignant tumor growth. In this study, we demonstrated that KC7F2, a HIF-1α inhibitor, inhibited HIF-1α-mediated VEGF production. Furthermore, HO-1 directly regulated HIF-1α production [17]. Therefore, HO-1 inhibitor such as ZnPP was demonstrated with capability in reduction of HIF-1α expression and VEGF levels in this study. We speculated that tumor inhibitory effect of ZnPP was partially due to decreasing HIF-1α expression through reducing HO-1 activity, and then consequently decreased tumor angiogenesis.

Tumor hypoxia often derived from tumor-lodging microenvironment in many solid tumors which receive limited amounts of oxygen supply promptly promotes the formation of new blood vessels. The advanced tumors exhibit large volume coupled with higher degree in angiogenesis [37]. In order to determine the inhibitory effects of ZnPP to angiogenesis, we selected adequate tumor size near to 100 mm^3^ for imaging angiogenesis after administration of ZnPP, in which was sufficient area for observing fluorescent signals on the location of implanted tumors. Besides, since the *in vivo* near-infrared AngioSense 680 EX fluorescent agent is a PEGylated large scaffold (250 kDa) belonging to a non-targeted tumor vascular fluorescent agents [38, 39], we utilized this agent to detect tumor angiogenesis. The results demonstrated that ZnPP significantly reduced the degree of tumor angiogenesis in the HCT-15-induced tumor xenografts.

It has been well known that hypoxia-induced HIF-1α mediates the down-stream signaling pathways for various forms of genes for response to tumor progression and invasion. Since elevated HIF-1α participates in tumor progression, HIF-1α is considered as one of tumor markers and can be useful as a targeted candidate for anti-tumor therapeutics. The therapeutic approaches by targeting to HIF-1α may impact several fields, including (a) reduction of HIF-1α synthesis, (b) accelerating degradation of HIF-1α, and (c) inhibiting the transactivation of the HIF-1α-mediated down-stream signaling events. In contrast to HO-1 inducer hemin increased HIF-1α levels, this study demonstrated that HO-1 inhibitor ZnPP reduced HIF-1α expression, indicating that HO-1 may regulate amounts of HIF-1α, and can be qualified as a targeted protein for developing anti-tumor therapeutics.

A previous study has reported that both ZnPP and hemin are an oxidative iron-binding porphyrin, inhibits CoCl_2_-induced HIF-1α expression through accelerating HIF-1α degradation [40]. However, this study revealed the induction of HIF-1α by hemin in the normal cultured condition, but only ZnPP reduced HIF-1α levels. The difference between hemin (iron) and ZnPP (zinc) is (a) in the function either to induce or inhibit HO-1 activity, and (b) the various forms of metals enclosed in the porphyrins. In addition to regulation of HO-1 activity, zinc metal may have other effects or biological roles to modulate the activity of HIF-1α in tumors [41, 42]. Our results revealed that ZnPP possessed higher inhibitory effects than hemin on HIF-1α expression and proliferation of tumor cells, suggesting that ZnPP is a therapeutic candidate for treatment of cancers.

## Conclusions

In conclusion, this study demonstrated that ZnPP is not only a HO-1 inhibitor, but also a potential anti-tumor agent inhibiting tumor proliferation. We found that ZnPP reduced HIF-1α expression in HCT-15 cells, and consequently inhibited hypoxia-mediated VEGF release. Moreover, we also demonstrated that ZnPP inhibited HCT-15 cell proliferation, and reduced tumor growth in the HCT-15-induced tumor xenografts as an ideal therapeutic agent. The angiogenesis level was decreased in the tumor exposed to ZnPP compared to the group treated without exposure of ZnPP. These findings may detail the ZnPP-mediated anti-tumor mechanism. Since the results revealed that VEGF was highly elevated in the sera of CRC patients using an ELISA assay, we speculated and suggested that ZnPP may be a potential therapeutic agent against CRC.
