# Correction: Blocking heme oxygenase-1 by zinc protoporphyrin reduces tumor hypoxia-mediated VEGF release and inhibits tumor angiogenesis as a potential therapeutic agent against colorectal cancer

**DOI:** 10.1186/s12929-025-01175-1

**Published:** 2026-07-27

**Authors:** Chun-Chia Cheng, Siao-Syun Guan, Hao-Jhih Yang, Chun-Chao Chang, Tsai-Yueh Luo, Jungshan Chang, Ai-Sheng Ho

**Affiliations:** 1 Institute of Nuclear Energy Research, Atomic Energy Council, Taoyuan, Taiwan; 2 Institute of Clinical Medicine, National Yang-Ming University, Taipei, Taiwan; 3 Division of Gastroenterology and Hepatology, Department of Internal Medicine, Taipei Medical University Hospital, Taipei, Taiwan; 4 Department of Internal Medicine, School of Medicine, College of Medicine, Taipei Medical University, Taipei, Taiwan; 5 Graduate Institute of Medical Sciences, School of Medicine, College of Medicine, Taipei Medical University, Taipei, Taiwan; 6 Division of Gastroenterology, Cheng Hsin General Hospital, Taipei, Taiwan; 7 Nursing Depratment, Kang-Ning University, Taipei, Taiwan

**Correction: Journal of Biomedical Science (2016) 23:18** 10.1186/s12929-016-0219-6

After the publication of our article, we became aware that a specific photograph illustrating the elevated levels of Vascular Endothelial Growth Factor (VEGF) in the serum of colorectal cancer patients [1] (illustrated in Fig. 1A–T) had previously appeared in Fig. 1A of a separate publication [2]. In order to provide clarity regarding the use of this recycled image, we would like to formally reference that publication within our article.

The image in question originates from a specimen collected from the colorectal cancer (CRC) patients involved in our study, which aimed to explore the roles of norepinephrine (NE) and the VEGF pathway in the progression of CRC. Our research findings, derived from this specimen, have been disseminated through two distinct journals: Oncotarget and the Journal of Biomedical Sciences. We acknowledge that we utilized the same batch of specimens across these different studies, leading to the concurrent publication of our results in various reputable journals. This approach reflects our commitment to advancing the understanding of CRC while ensuring that our work adheres to the highest standards of scientific integrity.

## References

[1] Cheng C-C, Guan S-S, Yang H-J, Chang C-C, Luo T-Y, Chang J, Ho A-S. Blocking heme oxygenase-1 by zinc protoporphyrin reduces tumor hypoxia-mediated VEGF release and inhibits tumor angiogenesis as a potential therapeutic agent against colorectal cancer. J Biomed Sci. 2016;23:18. 26822586 10.1186/s12929-016-0219-6 PMC4730655

[2] Ho AS, et al. Neutrophil elastase as a diagnostic marker and therapeutic target in colorectal cancers. Oncotarget. 2014;5:473–80. 24457622 10.18632/oncotarget.1631 PMC3964222

